# CRT-D or CRT-P?: the endless debate!

**DOI:** 10.1093/europace/euad285

**Published:** 2023-09-15

**Authors:** J Claude Daubert

**Affiliations:** Faculté de Médecine, Université de Rennes 1, 2 Av. du Professeur Léon Bernard, 35000 Rennes, France

The question raises important clinical and economic issues. We have to recognize that it has remained largely unanswered for 20 years.

The possible superiority of Cardiac Resynchronisation Therapy-Defibrillator (CRT-D) over Cardiac Resynchronisation Therapy-Pacemaker (CRT-P) could have been shown from the beginning of the 2000s if the analysis plan of the randomized phase III COMPANION trial had provided for a direct comparison between CRT-D and CRT-P. COMPANION randomized patients with a clinical indication for CRT into three arms as follows: CRT-D, CRT-P, and No-device therapy (control group).^1^ All patients were receiving optimal medical treatment for chronic heart failure (HF) as recommended at the time. Unfortunately, the analysis plan only provided for a head to head comparison between CRT-D and controls on the one hand, and CRT-P and controls on the other hand. No direct comparison between CRT-D and CRT-P had been planned although the size of the samples probably allowed it. The number of patients included in each of the two active arms was 600 vs. 300 in the control arm! The main objective of the study was a composite of all-cause death or hospitalization for HF analysed as 1st event delay. After a rather short median follow-up of 14 months, CRT-D and CRT-P provided a nearly similar benefit as compared with controls. Hazard ratio was 0.65 (95%CI 0.53–0.80) for CRT-P and 0.60 (95%CI 0.49–0.75) for CRT-D. The first hierarchical secondary endpoint of the trial was all-cause mortality at 12 months. The mortality rate was 19% in controls, 15% in the CRT-P arm, and 12% in the CRT-D arm. The difference was statistically significant for CRT-D compared with controls [HR 0.64 (0.48–0.86)], but not for CRT-P [0.76 (0.58–1.01)]. However, the results of a *post hoc* analysis with direct comparison of CRT-D vs. CRT-P were presented by M. Bristow, the study chairman, during the AHA’2005 Scientific Sessions (*abstract Circulation 2005; 112: II-673*) showing no additional survival benefit by CRT-D over CRT-P: HR 0.92 (0.82–1.35), *P* value = 0.33. The numerical difference was only 8%.

During the past two decades and despite clearly identified needs, no face-to-face comparison randomized clinical trial (RCT) could be successfully developed. We must therefore welcome the initiative of the Leipzig Heart Center to promote the ongoing ‘Re-evaluation of Optimal Re-synchronization Therapy in Patients With Chronic Heart Failure’ or RESET-CRT trial.^2^ This is an open-label RCT whose objective is to demonstrate that CRT-P is non-inferior to CRT-D in patients with chronic HF and an indication to CRT. The primary endpoint is all-cause mortality. The rationale of the RESET-CRT trial was based on the results of a national registry in Germany that included patients with *de novo* CRT device implantation from 2014 to 2019. Patients with implantable cardiac defibrillator (ICD) indication for secondary prevention of sudden cardiac death were excluded. Data from 847 CRT-P patients and 2722 CRT-D patients were analysed. Overall, 714 deaths (20%) were recorded during a median follow-up of 2.35 years. A higher cumulative incidence of all-cause death was observed in CRT-P patients in the initial unadjusted KM time-to-event analysis [HR 1.63 (95%CI 1.38–1.92)] but the difference became non-significant after adjustment for age [HR 1.13 (95%CI 0.95–1.35)] and after entropy balancing [0.99 (95%CI 0.81–1.20)]. No survival differences were found in different age groups.^3^ Recent information on ClinicalTrials.gov^3^ indicates that recruitment into the RESET-CRT trial had to be halted after inclusion of 959 patients due to lack of funding. It is therefore uncertain that the study will have sufficient power to conclude. It is therefore possible that we will still have to wait a long time before having robust clinical evidence, if it ever arrives!

Until a head to head comparison is available, we must rely on indirect clinical evidence from other large CRT morbidity and mortality trials, or from observational data. In the landmark CARE-HF study in NYHA III–IV HF patients,^4^ CRT *per se* was compared with medical therapy alone. CRT was shown to reduce the incidence of the combined primary endpoint by 37% [HR 0.63 (95%CI 0.51–0.77)], a similar proportion as COMPANION but also of all-cause mortality (secondary endpoint) by 36% [HR 0.64 (95%CI 0.48–0.85)]. Importantly, the mean duration of follow-up was twice as long in CARE-HF (29.4 months) than in COMPANION. During the 1-year extension phase of the study, CRT was also shown to reduce significantly the risk of cardiac sudden death [HR 0.54 (95%CI 0.35–0.84)].^5^ These data provide robust evidence that CRT *per se* improves survival in patients with moderate to severe HF.

Two large-scale RCTs assessed the value of adding CRT to ICD in HF patients with reduced ejection fraction (<30%). The RAFT trial^6^ included 1798 NYHA class II–III patients who were followed for a mean of 40 months. The MADIT-CRT trial^7^ included 1820 less severe patients (NYHA I–II) with a shorter mean follow-up duration (28 months). Although the clinical benefit was as expected for the primary composite endpoint in the two studies, the results were not consistent with the secondary endpoint of all-cause mortality. In RAFT, the mortality rate was significantly reduced with CRT-D: 20.8% vs. 26.1% [HR 0.75 (95%CI 0.62–0.91)] when there was no significant benefit in MADIT-CRT: 6.8% vs. 7.3% [HR 1.00 (95%CI 0.69–1.44)]. These data suggest that an additional survival benefit from adding CRT to ICD depends on the baseline severity of HF and the duration of follow-up in the trial.

In an individual patient meta-analysis of five randomized trials comparing CRT either with no active device or with a defibrillator (MIRACLE, MIRACLE-ICD, CARE-HF, RAFT, REVERSE), Cleland *et al.*^8^ showed that comparing patients assigned to CRT/CRT-D to the control group in the whole population, the HR for all-cause mortality was 0.66 (95%CI 0.57–0.77), and it was 0.65 (95%CI 0.58–0.74) for death or HF hospitalization. An exploratory subgroup analysis suggested no interaction between ICD and No-ICD patients for all-cause mortality. A different approach was proposed by Woods *et al.*^9^ through a network meta-analysis of individual patient data of the mortality effects of implantable cardiac devices (CRT-D, CRT-P, or ICD) in patients with HF and reduced EF. Data from 13 RCTs were selected for analysis. Unadjusted analyses found CRT-D to be the most effective treatment with a mean reduction of 19% in all-cause mortality compared with CRT-P, and 18% compared with ICD alone. Adjusted analyses showed that patients with QRS duration > 150 ms, LBBB morphology, age > 60 years, and female gender benefited more from CRT-P and CRT-D.

Overall, based on the data from RCTs and their meta-analyses, there is still no solid evidence of greater survival benefit by CRT-D compared with CRT-P in the overall population of patients with a clinical indication to CRT.

What about observational studies? In this issue of the journal, Veres *et al.*^10^ report the results of a literature review and meta-analysis based on 26 observational studies published over the past 20 years. A total of 20 are retrospective studies, and 6 correspond to prospective cohort studies. We can regret the absence in the list of the European CRT Survey that was a joint initiative of the Heart Failure Association (HFA) and the European Heart Rhythm Association (EHRA) of the ESC.^11^ Due to late publication, the data from the RESET-CRT registry were not taken into account.^3^ The methodological limits inherent in observational studies and their heterogeneity mean that the global results of the analysis essentially have an informative value on real-life practices and their evolution over time and therefore do not constitute real scientific evidence. The final analysis included 128 030 patients, 55 469 implanted with a CRT-P device, and 72 561 with a CRT-D. Patient baseline characteristics are not reported in detail. Within these limits, the results suggest an overall 15% reduction in all-cause mortality by CRT-D compared with CRT-P [HR 0.85 (95%CI 0.76–0.94)]. However, the difference was not statistically significant in certain subgroups of patients, in particular, older patients > 75 years [HR 0.95 (95%CI 0.79–1.15)] and patients with non-ischaemic cardiomyopathy [HR 1.08 (95%CI 0.96–1.21)]. Time-trend effects could be also observed in patients’ characteristics: CRT-P candidates were getting older, and the prevalence of ischaemic aetiology was increasing over years.

These subgroups are quantitatively important as illustrated by the data from the European CRT survey where 31% of patients were aged over 75 years.^11^ Following the data from the Veres study,^10^ this proportion has continued to increase over the years. Regarding aetiology, 40% of patients had a non-ischaemic cause.^11^ The observational data by Veres^10^ are consistent with those of the DANISH study,^12^ a RCT that randomized 1116 patients with symptomatic LV dysfunction (left ventricular ejection fraction < 35%) not caused by coronary artery disease into two arms as follows: ICD implantation or No-ICD. In both groups, 58% of the patients received CRT. During a median follow-up period of 67.6 months, 21.6% patients died in the ICD arm compared with 23.4% in the No-ICD arm. The difference was not statistically significant [HR 0.87 (95%CI 0.68–1.12)]. Prespecified subgroup analysis showed no interaction (*P* = 0.73) depending on whether patients had received CRT or not.

**Figure 1:**
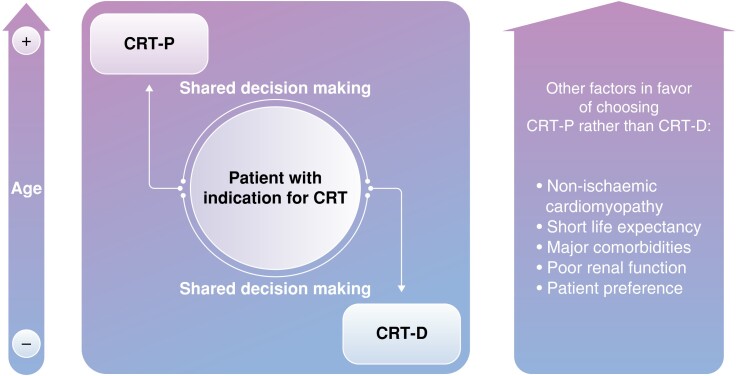
Inspired by Figure 10 from 2021’ESC guidelines on cardiac pacing and cardiac resynchronization therapy.^13^

Overall, Veres’ observational data tend to support international guideline recommendations.^13,14^ Due to the continued lack of robust clinical evidence, they do not provide strict recommendations on device selection, CRT-D or CRT-P in patients with a Class I or IIa indication for CRT. They only recommend shared decision-making taking into account individual patient characteristics (*Figure 1*), namely age and HF aetiology, but also common sense factors such as life expectancy, major comorbidities, poor renal function, and of course, patient preference.

**Funding**: None declared.
